# Transformation of low molecular compounds and soil humic acid by two domain laccase of *Streptomyces puniceus* in the presence of ferulic and caffeic acids

**DOI:** 10.1371/journal.pone.0239005

**Published:** 2020-09-18

**Authors:** Liubov I. Trubitsina, Alexander V. Lisov, Oxana V. Belova, Ivan V. Trubitsin, Vladimir V. Demin, Andrey I. Konstantinov, Anna G. Zavarzina, Alexey A. Leontievsky

**Affiliations:** 1 G. K. Skryabin Institute of Biochemistry and Physiology of Microorganisms, Russian Academy of Sciences (IBPhM RAS), Pushchino, Russia; 2 Faculty of Soil Science, Lomonosov Moscow State University, Moscow, Russia; 3 Faculty of Chemistry, Lomonosov Moscow State University, Moscow, Russia; Luleå University of Technology, SWEDEN

## Abstract

The two-domain bacterial laccases oxidize substrates at alkaline pH. The role of natural phenolic compounds in the oxidation of substrates by the enzyme is poorly understood. We have studied the role of ferulic and caffeic acids in the transformation of low molecular weight substrates and of soil humic acid (HA) by two-domain laccase of *Streptomyces puniceus* (SpSL, previously undescribed). A gene encoding a two-domain laccase was cloned from *S*. *puniceus* and over-expressed in *Escherichia coli*. The recombinant protein was purified by affinity chromatography to an electrophoretically homogeneous state. The enzyme showed high thermal stability, alkaline pH optimum for the oxidation of phenolic substrates and an acidic pH optimum for the oxidation of K_4_[Fe(CN)_6_] (potassium ferrocyanide) and ABTS (2,2′-azino-bis(3-ethylbenzothiazoline-6-sulfonic acid) diammonium salt). Phenolic compounds were oxidized with lower efficiency than K_4_[Fe(CN)_6_] and ABTS. The SpSL did not oxidize 3.4-dimethoxybenzoic alcohol and *p*-hydroxybenzoic acid neither in the absence of phenolic acids nor in their presence. The enzyme polymerized HA—the amount of its high molecular weight fraction (>80 kDa) increased at the expense of low MW fraction (10 kDa). The addition of phenolic acids as potential mediators did not cause the destruction of HA by SpSL. In the absence of the HA, the enzyme polymerized caffeic and ferulic acids to macromolecular fractions (>80 kDa and 10–12 kDa). The interaction of SpSL with HA in the presence of phenolic acids caused an increase in the amount of HA high MW fraction and a two-fold increase in the molecular weight of its low MW fraction (from 10 to 20 kDa), suggesting a cross-coupling reaction. Infrared and solution-state ^1^H-NMR spectroscopy revealed an increase in the aromaticity of HA after its interaction with phenolic acids. The results of the study expand our knowledge on the transformation of natural substrates by two-domain bacterial laccases and indicate a potentially important role of the enzyme in the formation of soil organic matter (SOM) at alkaline pH values.

## Introduction

Laccase (EC 1.10.3.2) is oxidoreductase that catalyzes the oxidation of its substrates by oxygen, which is reduced to water during the reaction. Laccase is widespread in nature, being produced by plants, free-living and symbiotic fungi and bacteria [1]. The laccase molecule contains an active site that includes four copper atoms, which are organized into three types of centers called type 1, 2, 3 [2]. There are two families of laccase which are distinguished by the three-dimensional structure of the molecule. Three-domain laccase is a monomeric protein consisting of three domains. The molecular weight of this type of laccase is usually 60–70 kDa [2], however, there are reports on the existence of an oligomeric form of the enzyme [3–5]. Three-domain laccases are widely distributed among plants, bacteria and fungi [6–8]. Another form of the enzyme, two-domain or “small” laccase, is a homotrimeric protein. The active site is located between the subunits of the enzyme, belongs to two subunits, and each monomer subunit consists of two domains [9]. The molecular weight of the subunit is about 35 kDa; the molecular weight of the native protein is about 110 kDa.

The substrates of laccase are various phenolic compounds, aromatic amines, aromatic dyes, metal ions [10–12]. As a result of substrate oxidation, the intermediate radicals and quinones are formed, which then undergo condensation or destruction reactions [13]. Laccase can metabolize both low molecular weight and high molecular weight compounds, e.g. lignin [14]. The catalytic properties of the two forms of laccase are different. Typically, three domain laccase catalyzes the oxidation of phenolic substrates with an optimum in the acidic pH range [2]. The optimal oxidation of phenolic compounds by two-domain laccase is in the alkaline pH range [15]. Two-domain laccase oxidizes phenolic compounds less efficiently than three-domain laccase [16, 17].

A separate group of reactions catalyzed by laccase involve the participation of low molecular weight redox mediators [2]. Mediators are low molecular substances that are oxidized by laccase to intermediate radicals, which then oxidize various compounds independently of the enzyme [18]. The presence of mediators extends the oxidation potential of laccase. Artificial and natural compounds are known to be mediators for laccase [19, 20]. Mediators of natural origin are products of lignin breakdown (e.g. syringyl and vanillyl phenols), plant secondary metabolites (e.g. vanillin, p-coumaric acid), as well as fungal metabolites such as 3-hydroxyanthranillic acid [1]. For example, 3-hydroxyanthranillic acid allowed fungal laccase to oxidize non-phenolic units of lignin [21]. Methyl syringate promoted lignin degradation by the enzyme [22]. Syringyl and vanillyl phenols were effective mediators in the degradation of recalcitrant synthetic dyes by laccase [20]. Some phenolic mediators facilitated the oxidation of polycyclic aromatic hydrocarbons by laccase [23]. However, all these reactions were carried out with three-domain laccase, and not with two-domain laccase. The role of mediators in the substrate oxidation by two-domain laccase is poorly understood.

A large group of natural phenolic compounds are humic substances (HS). They are ubiquitous in the environment and are described as dark-colored products of the oxidative transformation of organic, primarily plant, residues in soil [24]. Biotransformations include both biodegradation and oxidative coupling reactions [24–26]. The origin, biochemical stability, macromolecular structure, and existence of HS as a distinct class of organic compounds is currently under debate [27–29]. Nevertheless, dark-colored organic matter is specific to soils and its alkali-extractable fraction (humic and fulvic acids) represent 30–50% of soil organic carbon, depending on soil type [30]. It has been shown that three-domain fungal laccase can polymerize and depolymerize humic acids (HA) both *in vivo* and *in vitro* [4, 31, 32]. Two-domain bacterial laccase polymerized humic acids and their low- and high-molecular weight fractions [33]. Thus, both forms of laccase play a potentially important role in the transformation of components of soil organic matter (SOM). However, it is unclear whether two-domain bacterial laccase can catalyze the depolymerization of HA. It has been reported that natural phenolic mediators possibly enhance lignin decomposition by two-domain laccase [34]. The role of phenolic compounds in HA degradation by two-domain laccase has not been studied so far.

The aim of this work was to study the role of natural phenolic compounds—ferulic acid (FA) and caffeic acid (CA)–in the transformation of low molecular weight substrates and soil humic acid by two-domain bacterial laccase. Ferulic and caffeic acids have been chosen for the study since they are widely distributed in nature, exist in a free form in the plant cell wall [35–37] and in the soil solutions as product of lignin breakdown [38]. A new member of the family of two-domain laccases, the enzyme from soil bacterium *Streptomyces puniceus* (designated as SpSL) was used. Bacteria of the genus *Streptomyces* are widely distributed in soils and are able to transform HS [39]. However, the enzyme systems involved in this process are poorly understood. The participation of peroxidase as well as cell-associated enzymes has been suggested to catalyze HA degradation [40]. Soil bacterium *S*. *puniceus* is known to grow on humic acid-containing HV-agar [41]. Enzymatic systems of *S*. *puniceus* that transform HS have not been studied so far. Therefore, the gene encoding two-domain laccase was cloned from *S*. *puniceus*, over-expressed in *E*. *coli*, the enzyme has been purified and its properties were studied. Therefore, the gene encoding two-domain laccase was cloned from *S*. *puniceus*, over-expressed in *E*. *coli*, after which the resulting enzyme was purified and its properties were studied.

We have shown that ferulic acid and caffeic acid did not act as mediators of the oxidation of 3.4-dimethoxybenzoic alcohol and of *p*-hydroxybenzoic acid by two-domain laccase at alkaline pH values. Phenolic acids also did not act as mediators of soil HA decomposition by bacterial laccase. Instead, the interaction of the enzyme with HA in the presence of phenolic acids caused the formation of polymers of higher molecular weight than have been formed from HA or from each of the phenolic acids in the presence of laccase. The results of the study strongly suggest the participation of two-domain laccase in the cross-coupling reactions between HA and phenolic acids. Such reactions may be of significance for the processes of SOM formation at alkaline pH values.

## Materials and methods

### Microorganism, the protein expression and purification

Strain *S*. *puniceus* VKM Ac-579 was obtained from the All-Russian collection of microorganisms (http://www.vkm.ru/index.htm). All procedures for bacteria cultivation, two-domain laccase’s gene cloning and the protein expression at low isopropyl β-D-1-thiogalactopyranoside (IPTG) concentration and weak aeration were identical to those previously described [17]. The gene was obtained from *S*. *puniceuss* DNA using PCR and cloned in pAL-TA vector. The primers for PCR, 5’-ATGGACCGAAGGACC and 3’-TCAGTGCTGGTGCC, were constructed on the basis of the sequence of two-domain laccase from *S*. *puniceus* NRRL ISP-5083 (NCBI Reference Sequence of protein: WP_030190946.1). Further the gene was amplified from pAL-TA by PCR with primers 5’-AGTGGATCCGAGAAGCCGCCCCG and 3’-TCAAAGCTTTCAGTGCTGGTGCC and was cloned into pQE-30 expression plasmid. Expression of the protein was done in *E*. *coli* M15 (pREP4). The protein purification was done in one stage by nickel affinity chromatography on HisTrapp 5 ml column (GE Healthcare, USA). The purification conditions were identical to [17]. After the chromatography stage, the protein was dialyzed against 20 mM Tris-HCl buffer (pH 8.5).

### The protein characterization

The protein sequence was analyzed using BLAST (http://blast.ncbi.nlm.nih.gov/Blast.cgi) and InterPro Scan (https://www.ebi.ac.uk/interpro/) services. The pH optimum, pH stability, the optimum of temperature for the activity, thermal stability, the UV-Vis absorption spectrum, and the molecular weight of native and denaturated protein were determined as it was described earlier [17]. Calculation of the apparent kinetic constants was performed by a nonlinear regression of the data using Sigma Plot 11.0 software.

### Ferulic and caffeic acids and humic acid preparation

Commercially available phenolic compounds (Sigma-Aldrich, USA) were used. Humic acid from soddy-podzolic soil (Stagnic Retisol, Loamic, Humic, according to WRB 2015) was used. Extraction, purification and characterization of HA (preparation HART) was as described earlier [30]. The ash content of the preparation was 2.6%.

### Reactions of two-domain laccase with low molecular weight phenolic and non-phenolic compounds in the presence of FA and CA

Oxidation of the non-phenolic compound 3.4-dimethoxybenzoic alcohol (3.4 DMBA) and of the phenolic compound *p*-hydroxybenzoic acid (*p*-HDB) by SpSL in the presence of ferulic and caffeic acids was carried out in 20 mM Tris-HCl buffer (pH 8.3) at 30°C during 48 h. The concentration of 3.4 DMBA and p-HDBA was 1 mM, the concentration of ferulic and caffeic acids was 3 mM, the activity of two-domain laccase was 0.5 U/ml. The concentration of compounds was determined by HPLC on Vision HT C18 column (Grace, USA). Elution was carried out by linear gradient from 10% methanol / 90% water to 100% methanol in 20 minutes. The peaks were detected using a DAAD detector.

### Reactions of two-domain laccase with humic acid in the presence of FA and CA

#### Reactions of SpSL with HA in the presence of FA and CA

Reactions were performed in 20 mM Tris-HCl buffer (pH 8.3) at 30°C during 48 h. The concentration of HA was 1 mg/ml, the concentration of phenolic acids was 3 mM, the activity of two-domain laccase was 0.5 U/ml, the volume of the reaction mixtures– 50 ml. The laccase activity was routinely determined by the rate of 2.2-azino-bis-(3-ethylbenzthiazolin-6-sulfonate) (ABTS) oxidation. The reaction mixture contained 1 mM ABTS in 20 mM Na-acetic buffer pH 5.0 and the enzyme preparation. The absorption was monitored at 420 nm (ε420 = 36 000 M^-1^ × cm^-1^) [42].

#### Gel-filtration

The molecular weight distributions of the HA and phenolic acids and the products of their reaction with two-domain laccase were obtained in conditions similar to those previously described [33]. The column filled with Sephadex G-75 gel was used. The column size was 2 × 42 cm (diameter × length), the 25 mM Tris-HCl buffer (pH 8.2) with an addition of 0.02 M NaCl and 0.01% SDS was used as an eluent. The void volume (V_0_) of the column was determined as an elution volume of Blue Dextran 2000, total void volume (V_t_) was determined as an elution volume of 0.5 M NiSO_4_ (detection by a conductometer). The column was calibrated using molecular weight markers: albumin from bovine serum (66 kDa), carbonic anhydrase from bovine erythrocytes (29 kDa), and cytochrome c from horse heart (12.4 kDa).

#### Spectroscopic characterization of the reaction products

The reaction mixtures were acidified to pH 2 with concentrated HCl. The precipitate was washed with distilled water on the centrifuge until the pH of distilled water was reached. The pellet was then dried in the open air.

#### Infrared spectroscopy

Infrared spectra were recorded using KBr pellets on a Bruker Tensor 27 spectrophotometer (Germany). The amount of KBr for the preparation of the pellet was 200 mg and the amount of sample was 3 mg, except for initial CA and FA (1 mg). Prior to the preparation of the pellet, its components were dried under vacuum at 60°C for 24 hours and then thoroughly mixed by grinding with a pestle in agate mortar.

#### Solution-state ^1^H NMR spectroscopy

Solution state ^1^H NMR spectra were acquired using a Bruker Avance 400 NMR spectrometer operating at 400 MHz proton frequency. The conditions of registration of the spectra and preparation of the samples for measurements in DMSO-d6 were as described in [43]. Briefly, a weight of 10–15 mg of a sample was dissolved in 0.75 ml of DMSO-d6 of 99.99% isotope purity (Aldrich), centrifuged and moved into a 5-mm tube for NMR. The acquisition time and relaxation delay were 1.66 s and 1 s, respectively. The number of scans for each NMR experiment amounted to 30–100. The spectra acquired were Fourier-transformed and baseline corrected with the use of MestReC software.

## Results

### Properties of two-domain laccase

**Fig 1:**
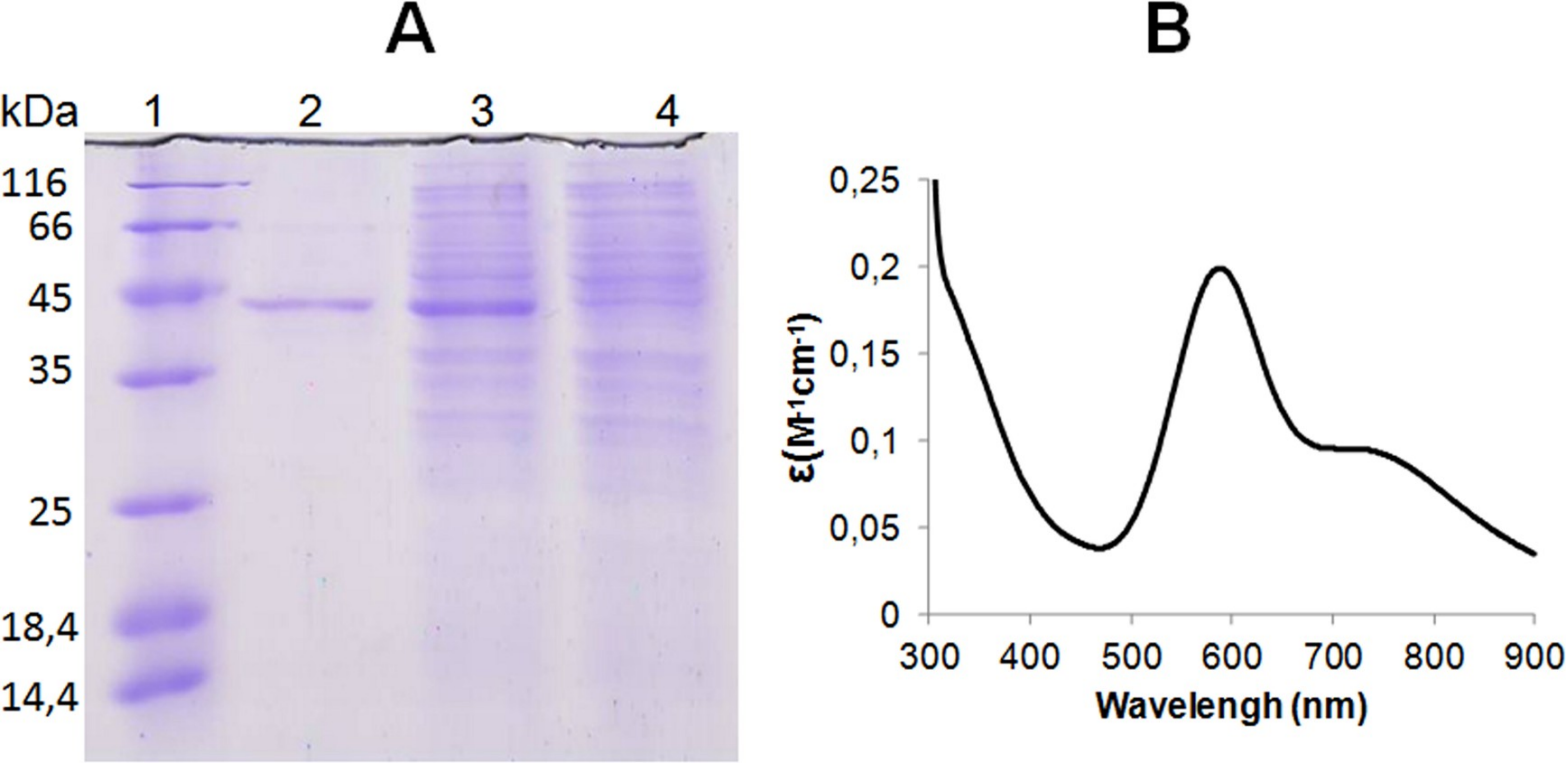
SDS-PAGE (A) and absorption spectra of SpSL (B). (A): 1, molecular weight markers; 2, SpSL preparation after a HisTrapp 5-ml column; 3, *E*. *coli* culture with IPTG; 4, *E*. *coli* culture without IPTG.

The cloned bacterial gene encoded a protein of 344 amino acid residues in length and had a completely identical sequence with the gene sequence from databases (WP_030190946.1). The protein was designated as SpSL. As a part of the protein sequence, there were two cupredoxin domains responsible for the binding of copper ions: they were located between 38 and 184 and between 190 and 320 amino acid residues. SpSL contains the copper-binding motifs, i.e. ten histidines and one cysteine. The protein had a TAT signal peptide from 1 to 34 amino acid. According to the SDS-PAG electrophoresis (Fig 1A) the molecular weight of the monomeric protein was 40 kDa, which was consistent with the molecular weight of the protein, calculated on the basis of the protein sequence (37.1 kDa). The molecular weight of the native protein, obtained by gel filtration, was 110 kDa, which indicates that SpSL was trimeric. After purification, the enzyme preparation was blue.

**Fig 2:**
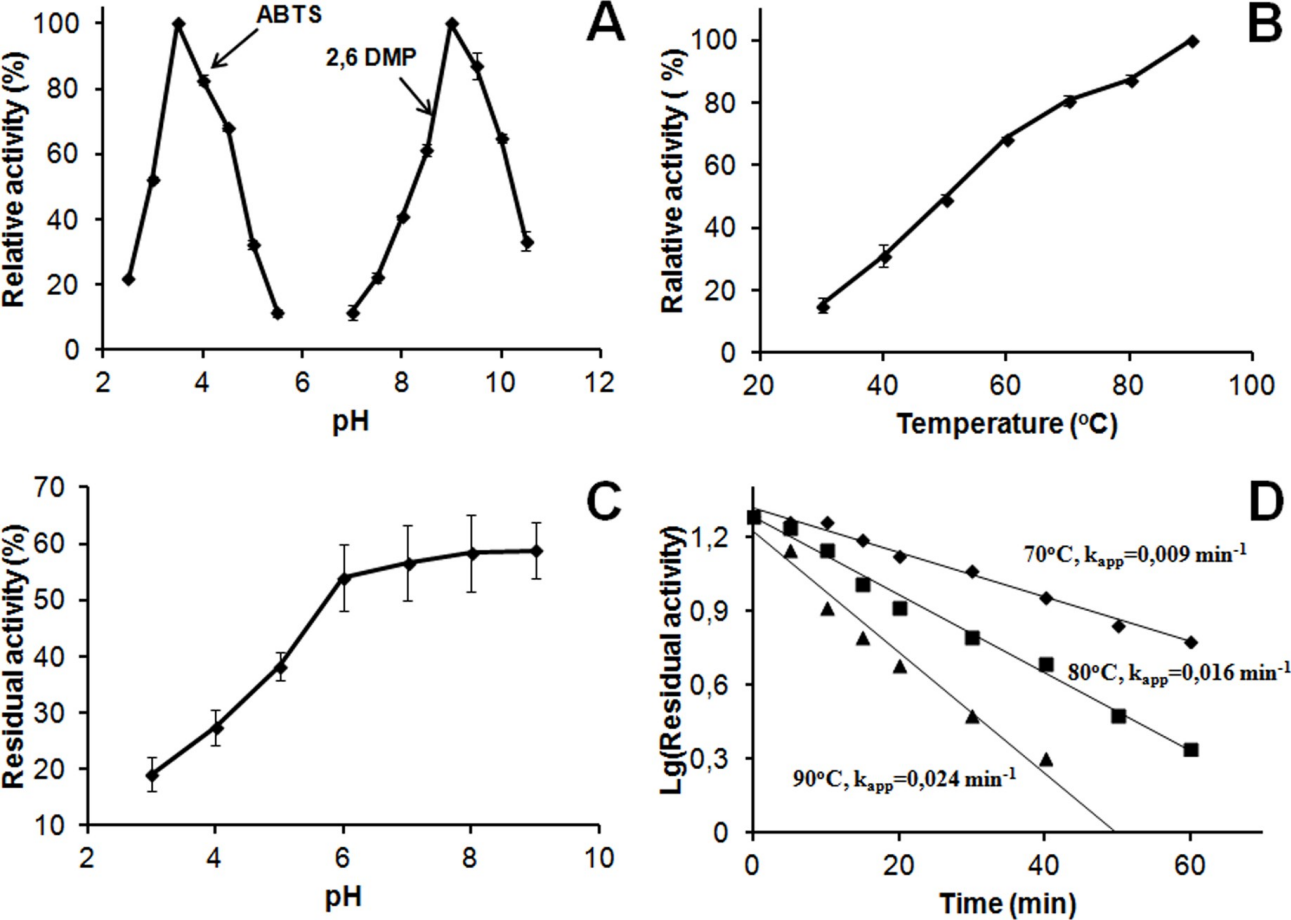
Properties of SpSL. (A) pH optimum of the enzyme with ABTS and 2,6-dimethoxyphenol;(B) effect of temperature on SpSL activity; (C) pH stability; (D) thermal stability at 70, 80 and 90°C.

The absorption spectrum of SpSL had a maximum at 600 nm and a shoulder at 340 nm (Fig 1B). SpSL had high thermal stability, alkaline pH optimum for the oxidation of phenolic substrates and stability in the alkaline pH region (Fig 2, the S1 Enzyme properties). The enzyme oxidized K_4_[Fe(CN)_6_] and ABTS more efficiently than 2-methoxyphenol and 2,6-dimethoxyphenol (Table 1).

**Table 1:** Kinetic parameters for the oxidation of phenolic and nonphenolic substrates by two-domain laccase of *S*. *puniceus*.

Substrate	*K*_m_, mM	*k*_cat_, sec^-1^	*k*_cat_/*K*_m_, mM^-1^ × sec^-1^
**K**_**4**_**[Fe(CN)**_**6**_**]**	0.87 ± 0.04	39 ± 0.7	44.8
**ABTS**	0.37 ± 0.021	24.3 ± 0.26	65.6
**2,6-Dimethoxyphenol**	1.15 ± 0.1	3.4 ± 0.064	2.95
**2-Methoxyphenol**	5.7 ± 0.25	0.85 ± 0.03	0.15

### Reactions of two-domain laccase with low molecular weight substrates in the presence of FA and CA

We have tested whether FA and CA can serve as mediators in the oxidation of phenolic and non-phenolic compounds otherwise not oxidizable by SpSL. We have found that after 48 hours of the reaction, there was no decrease in the amount of 3.4 DMBA and *p*-HDB in mixtures with phenolic acids, while FA and CA were completely oxidized. No interaction of oxidized FA and CA with *p*-HDB was observed.

### Interaction of the humic acid with SpSL in the presence of FA and CA

**Fig 3:**
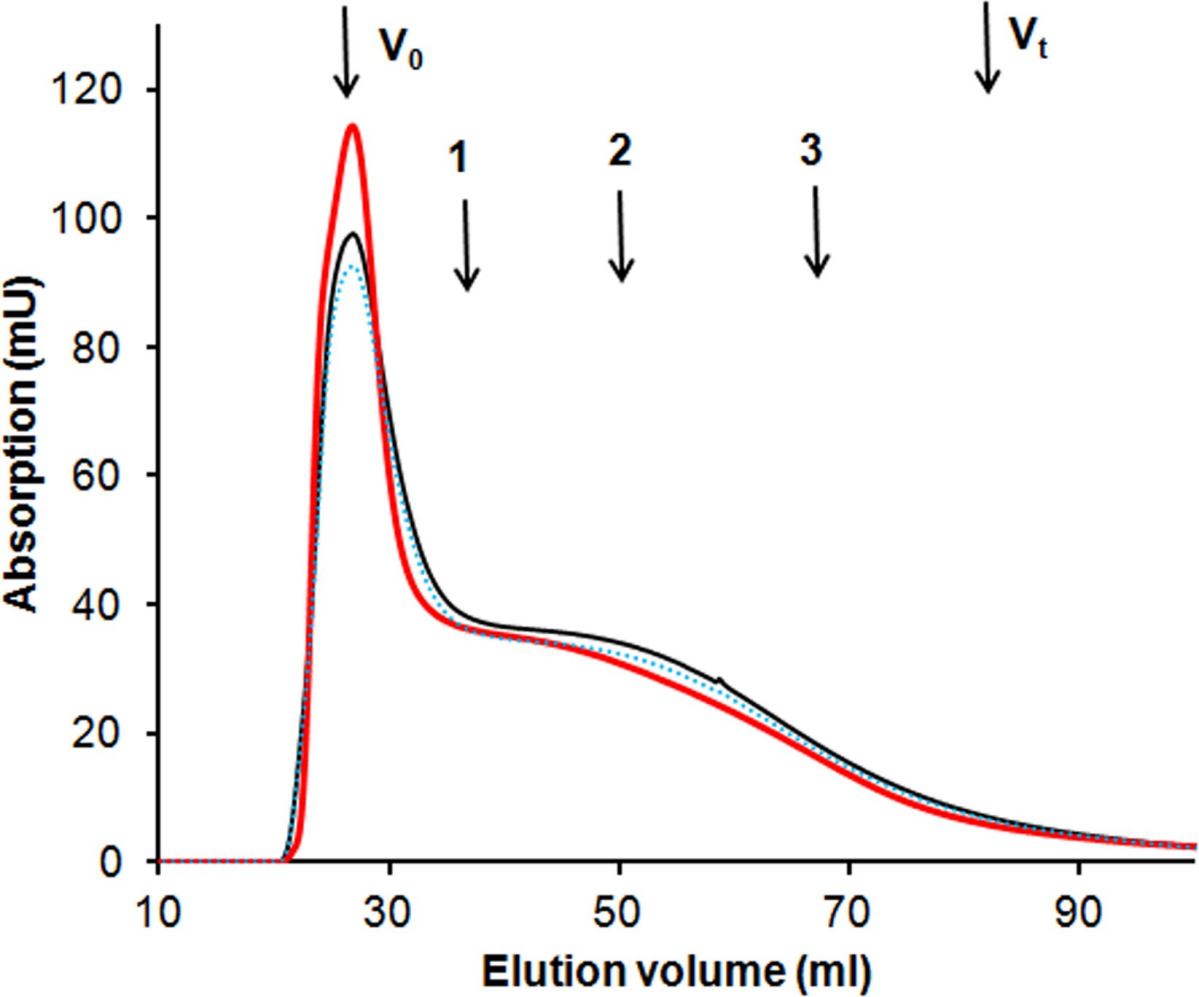
Gel-filtration profiles for the transformation of sod-podzolic humic acid (HA) by SpSL. Solid red line–HA after interaction with SpSL, thin black line–initial HA, dotted blue line–control with inactivated SpSL. Arrows indicate the void volume (V_0,_ >80 kDa), the total void volume (V_t_, <5 kDa), 1—bovine serum albumin (66 kDa), 2—carbonic anhydrase (29 kDa), cytochrome c 12.4 kDa).

In order to study the role of FA and CA in HA transformation by two-domain laccase, the reactions of HA with SpSL were performed in the presence and absence of CA and FA. The initial sod-podzolic HA consisted of two fractions–high molecular weight (MW> 80 kDa) and low molecular weight fraction (peak average MW 10 kDa). HA polymerized in the presence of SpSL (Fig 3). The amount of high molecular weight fraction increased and the amount of low molecular weight fraction decreased (Fig 3, the S1 Gel-filtration).

**Fig 4:**
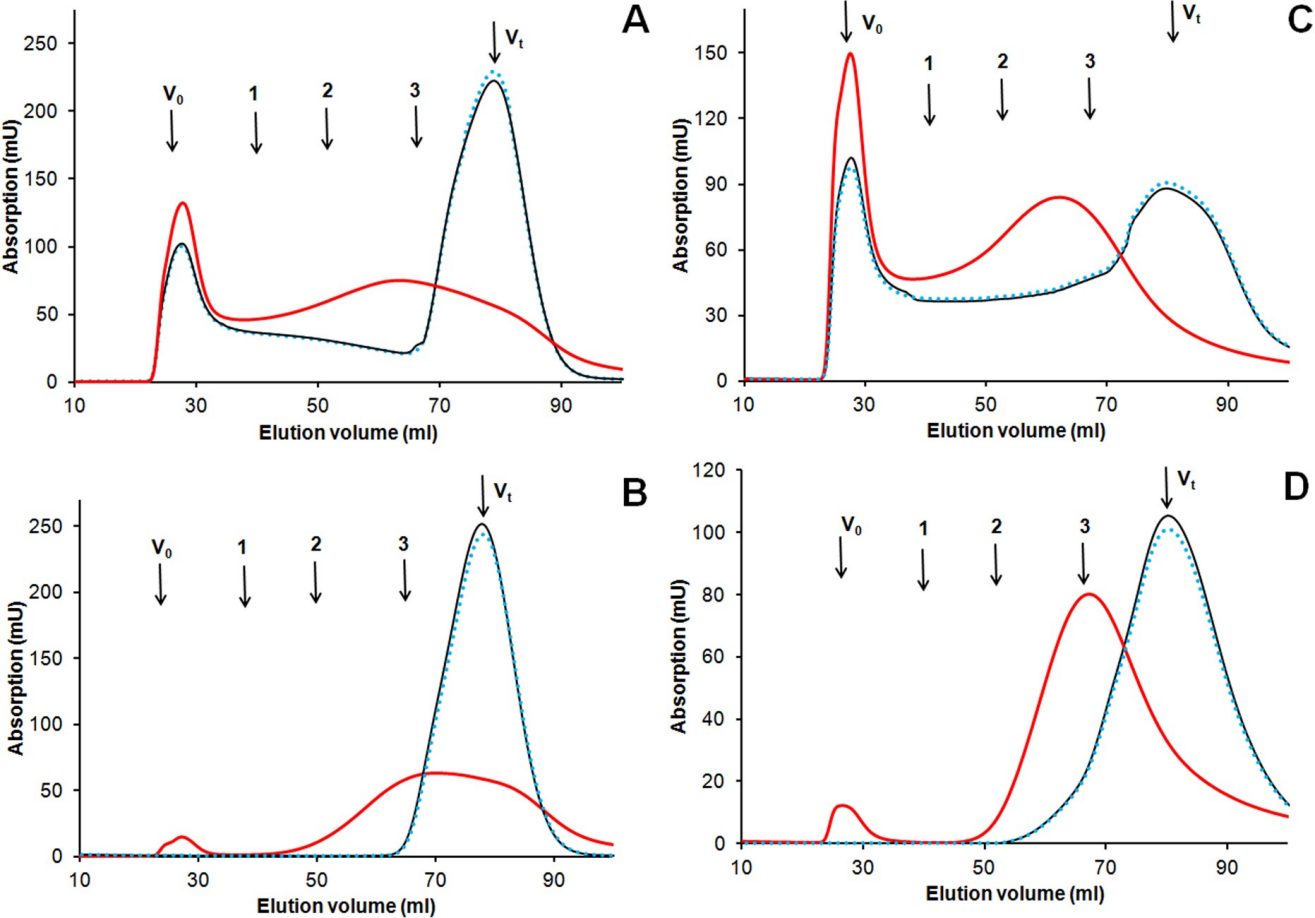
Gel-filtration profiles for transformation of ferulic acid, caffeic acid and their mixtures with HA by SpSL. (A) ferulic acid and humic acid; (B) ferulic acid; (C) caffeic acid and humic acid; (D) caffeic acid. Solid red line–sample after interaction with SpSL, thin black line–initial sample, dotted blue line–control with inactivated SpSL. Arrows indicate the void volume (V_0,_ >80 kDa), the total void volume (V_t_, <5 kDa), 1—bovine serum albumin (66 kDa), 2—carbonic anhydrase (29 kDa), cytochrome c 12.4 kDa).

The interaction of the enzyme with HA in the presence of phenolic acids did not result in a depolymerization reaction. Instead, an increase in the amount of HA high MW fraction occurred (Fig 4A and 4C, the S1 Gel-filtration), as well as an increase in the peak average MW of the low molecular weight fraction—from 10 kDa to 20 kDa in the presence of FA (Fig 4A) and from 10 kDa to 22 kDa in the presence of CA (Fig 4C).

We have tested whether the oxidation and polymerization of FA and CA by SpSL could contribute to the observed increase in the MW of the HA. Each of the phenolic acids was polymerized by SpSL. During the oxidation of FA, a small amount of high molecular weight polymer (MW about 80 kDa) and a large amount of lower MW polymer with a peak average MW of about 10 kDa were formed (Fig 4B). Caffeic acid was oxidized in a similar way, with the formation of a small amount of a polymer with MW>80 kDa and a large amount of polymer with a peak average MW of 12 kDa (Fig 4D). No formation of 20–22 kDa fraction occurred.

**Fig 5:**
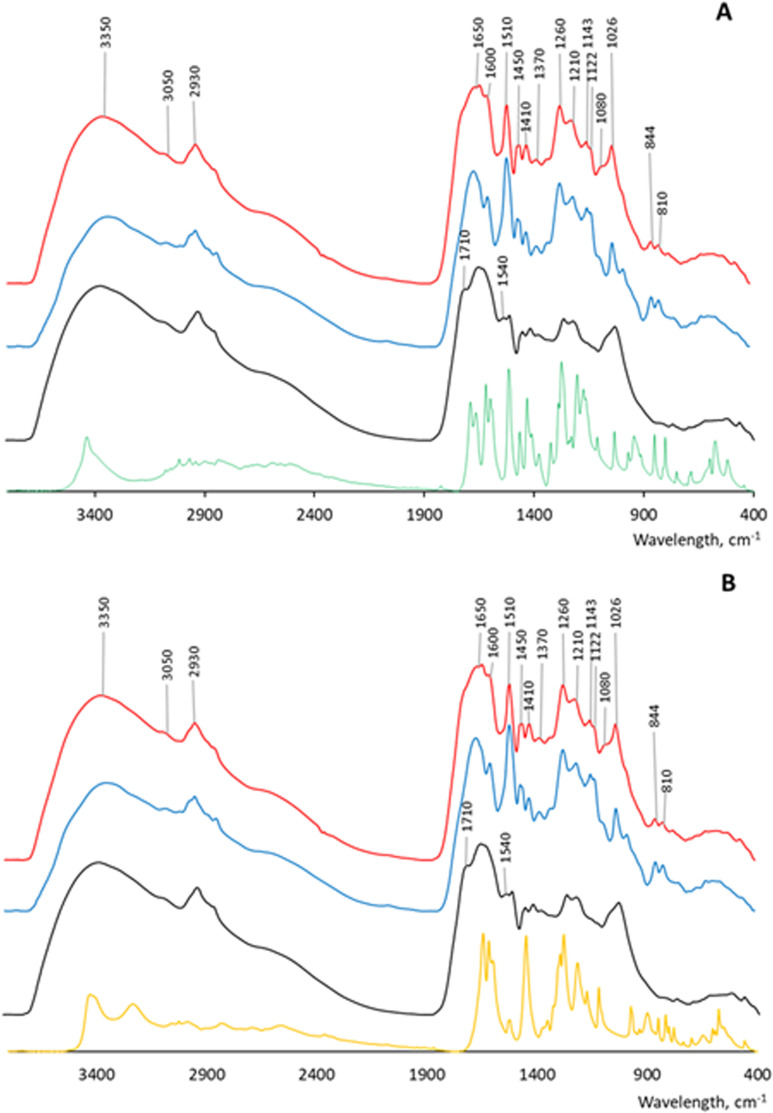
Infrared spectra (KBR-technique) of the products of the interaction of two-domain laccase of *S*.*puniceus* (SpSL) with humic acid (HA) in the presence and absence of: (A) ferulic acid; (B) caffeic acid. Black line–initial humic acid; green line–initial FA; yellow line–initial CA; blue line—phenolic acids after interaction with SpSL; red line–HA-phenolic acid mixtures after interaction with SpSL. The spectrum of the HA after interaction with SpSL was identical to the spectrum of initial HA and is given in (S1 Spectra).

In spectroscopic studies, we used acid precipitation to obtain the products of the reaction of SpSL with HA and phenolic acids. The yield of the dry samples after the reaction with the enzyme was approximately as follows: HA– 20 mg, ferulic acid polymer– 23 mg, caffeic acid polymer– 18 mg, HA with ferulic acid– 35 mg, HA with caffeic acid– 41 mg. The IR spectra are shown in Fig 5.

Absorption bands and corresponding peak assignments are summarized in Table 2. The common feature of all spectra is a broad and intense band at 3200–3400 cm^-1^, attributed to the stretching of hydrogen-bonded OH groups, and a shoulder at 3050 cm^-1^, attributed to aromatic C-H stretching. The weak intensity of this band may be due to substitution in the aromatic ring or due to overlap from the broad band of the OH stretching. All spectra possess a band at 2930–2914 cm^-1^ and a shoulder at about 2840 cm^-1^ ascribed to asymmetric and symmetric stretching of aliphatic C-H groups. The former band is the weakest in poly-CA. The spectrum of the initial HA contains a band at 1710 cm^-1^ (C-O of COOH groups) characteristic of the H-form of the HA preparations, and expressed as a shoulder, because it is almost completely overlapped by the band at 1650 cm^-1^.

**Table 2:** Absorption bands and their relative intensity in the IR spectra of the HA, polyphenolic acids and HA-phenolic acid polymers^a^.

Wavenumber, cm^-1^	HA	Poly-FA	HA-FA	Poly-CA	HA-CA	Bands assignments
**3200–3400**	s	s	s	s	s	Aromatic О–Н stretching, hydrogen-bonded OH
**3050**	vw	-	vw	vw	vw	Aromatic С–Н stretching
**2920–2950**	s	m	m	w	m	Aliphatic С–Н stretching
**1710**	shoulder	-	-	-	-	С = О stretching of COOH, aldehydes and ketones
**1650–1630**	s	s	vs	s	vs	C = O stretching of amide groups (amide I band);
						C = O of quinone and conjugated ketones
**1600**	-	m	shoulder	-	-	Aromatic C = C stretching, COO^−^symmetric stretching
**1530**	vw	-	-	-	-	N–H deformation and С = N stretching (amide II band)
**1510**	vw	s	vs	vs	m	Aromatic C = C stretching
**1450–1440**	w	s	s	m	-	Aromatic C = C stretching, C–H assymetric bending
**1420**	w	m	s	-	-	C–O–C stretching of methoxy groups
**1370**	vw	m	w	m	vw	O–H deformation and C–O stretching of phenolic groups
						COO antisymmetric stretching
						aliphatic C–H bending
**1260**	m	s	s	vs	s	С–О stretching and OH deformation of COOH,
**1210**	m	m	m	-	m	С–О stretching of aryl ethers and phenols
**1140**	-	m	m	-	-	C–OH stretching of aliphatic OH
**1120**	-	vw	shoulder	vw	vw	C–OH stretching of aliphatic OH
**1080**	shoulder	-	w	-	sh	С–О–C stretching of ethers
**1030–1020**	s	s	s	w	s	С–О stretching of polysaccharide-like substances
**975–775**	-	m	w	m	w	Out of plane bending of aromatic C–H

^a^Bands identification is according to Senesi et al., 2003; https://www.sigmaaldrich.com/technical-documents/articles/biology/ir-spectrum-table.html

vs–very strong, s–strong, m-mediun, w–weak, vw–very weak, sh—shoulder

The very intense band at 1650–1630 cm^-1^ (C = O stretching) can be attributed to quinones and conjugated ketones in poly-FA and poly-CA, while in HA-containing samples it can be also ascribed to the C = O of amide groups (amide I band). The presence of N-containing compounds in the HA structure is confirmed by the weak bands at 1530 cm^-1^ (amide II band) and at 1420 cm^-1^ (amide III band). Similar spectra of HAs with a high contribution from N-containing compounds are known from literature [24, 44]. The small peak at 1600 cm^-1^ in the poly-FA can be ascribed to aromatic C = C stretching and COO^-^ symmetric stretching. The peak is also present as a shoulder in the HA-FA. The band at 1510 cm^-1^, very weak in the initial HA and intense in the phenolic acid polymers and their associations with HA (especially in the HA-FA, Fig 5A), can be ascribed to aromatic C = C stretching in phenolic acids. The peak at 1450 cm^-1^ is also ascribed to aromatic C = C stretching. It is intense in the phenolic acid polymers and in HA-FA, while weak in HA and absent in HA-CA (Fig 5A and 5B). The peak at 1420 cm^-1^ can be ascribed to methoxy groups, because it is of weak intensity in the HA, intense in FA-containing samples and is absent in poly-CA and HA-CA. The band at 1380 cm^-1^ of a different intensity is attributed to phenolic groups and COO- antisymmetric stretching (Table 2). The bands at 1260–1210 cm^-1^ (present as one peak in poly-CA) are attributed to C-O stretching and OH deformation of the COOH group, as well as to C-O stretching of aryl ethers and phenols. These bands are weaker in the HA than in phenolic acid polymers and in their associations with HA, suggesting contribution of ether linkages in the polymerization reaction. The band at 1080 cm^-1^, present as a shoulder in HA-FA, is characteristic of ethers (C-O-C group stretching). In general, the data of IR spectroscopy show high contribution of poly-FA and poly-CA into the products of HA interaction with phenolic acids in the presence of SpSL. The enzyme most likely crosslinks poly-FA and poly-CA to HA to form a heterogeneous polymer. The increased intensity of the peaks at 1650, 1510, 1450, 1260 cm^-1^ in the HA-CA and HA-FA in comparison to the initial HA preparation shows the increase in aromaticity of the HA after its interaction with two-domain laccase in the presence of phenolic acids (Fig 5).

**Fig 6:**
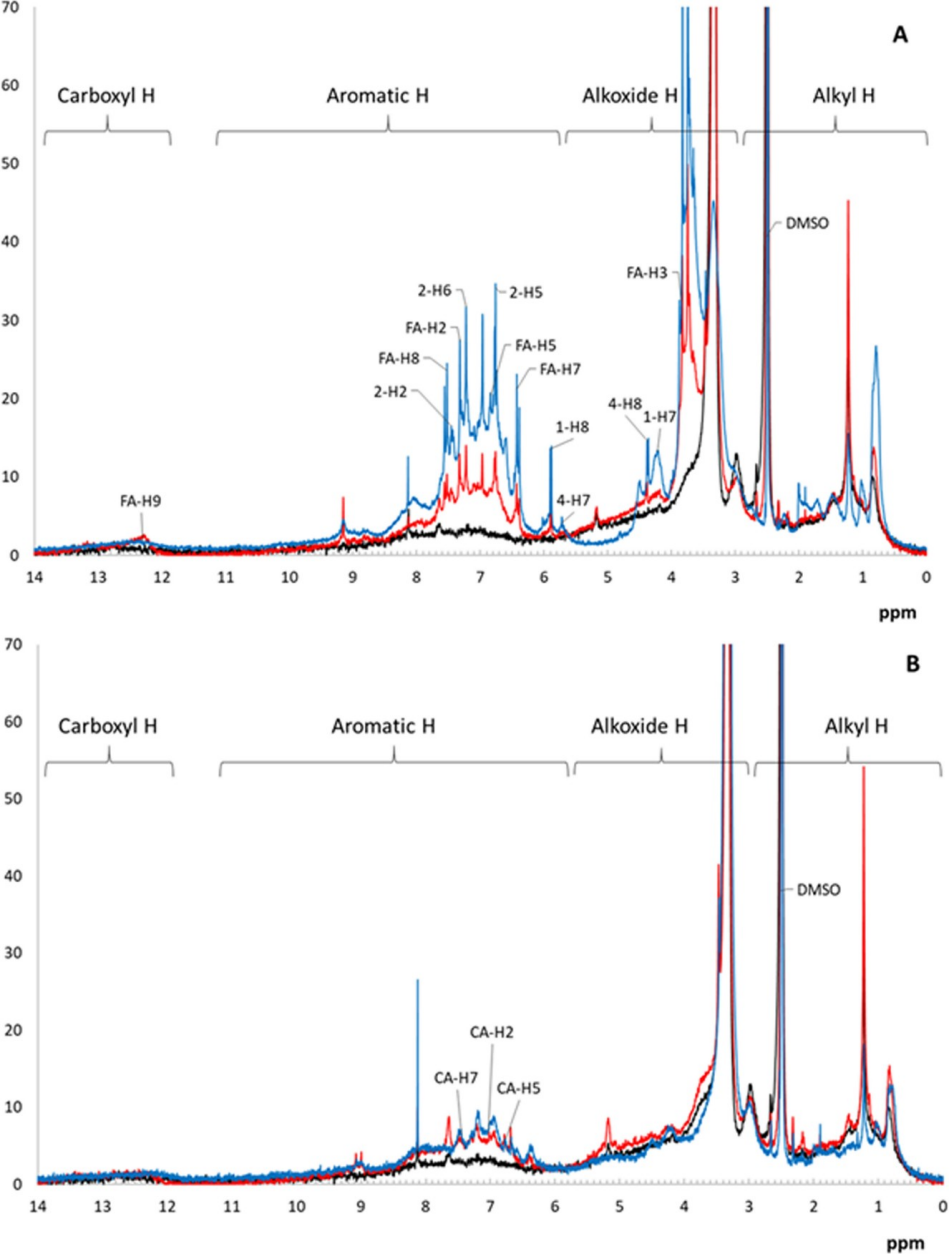
Solution-state ^1^H-NMR spectra (in DMSO-d6) of the products of the interaction of two-domain laccase of S.*puniceus* (SpSL) with humic acid (HA) in the presence and absence of: (A) ferulic acid (FA); (B) caffeic acid (CA). Black line—humic acid after interaction with SpSL; blue line—phenolic acids after interaction with SpSL; red line–HA-phenolic acid mixtures after interaction with SpSL. Individual signals assigned to phenolic acids are given according to Spectral Database for Organic Compounds (https://sdbs.db.aist.go.jp/). Signals assigned to the protons of FA dimers and designations of compounds are given according to He et al (2020): 1 –FA dilactone, 2–8-8-o diferulic acid, 4 –trans-lactone acid. Structures of compounds 1, 2 and 4 are given in (He et al., 2020).

The ^1^H NMR spectra of the reaction products of HA and phenolic acids with SpSL are shown in Fig 6. The spectral region from 0 to 2.8 ppm is attributed to the hydrogen of alkyl chains. The sharp peak at 2.5 ppm corresponds to residual protons of the solvent (DMSO-d6). The spectral area from 2.8 to 5.5 (5.8) ppm is attributed to the hydrogen of alkoxide structures and alkohol OH-groups, the area at 5.5(5.8)-11.0 ppm corresponds to aromatic hydrogen atoms. Lastly, the wide spectral band from 12 to 14 ppm corresponds to hydrogen of carboxylic groups. In general, the ^1^H NMR spectrum of HA is typical for those of humic materials and is characterized by broadband spectral signals. In constrast, the spectra of polyphenolic acids as well as the spectra of HA-FA and HA-CA contain sharp signals over the broadband background associated with protons in the structures of individual low molecular weight compounds (Fig 6).

The sharp signals at 3.83, 6.39, 6.82, 7.32, 7.52, 12.2 ppm in poly-FA and HA-FA are assigned to the protons of FA (Fig 6A), while the signals at 6.75–6.78, 7.02 and 7.45 in poly-CA and HA-CA are assigned to the protons of CA (Fig 6B). The sharp signals at 4.20, 4.40, 5.70, 5.90, 6.76, 7.22, 7.43 ppm in the spectra of poly-FA and HA-FA are assigned as protons belonging to dimers of FA (Fig 6A). The spectra of the HA after the reaction with phenolic acids in the presence of SpSL look quite similar to the spectra of poly-CA and poly-FA (Fig 6), indicating the modification of HA with products of phenolic acids oxidation. The contribution of poly-FA into HA structure (Fig 6A) was much more pronounced than that of poly-CA (Fig 6B). Poly-FA contributed largely to the increase in signals of aromatic protons in the HA and signals at 3.5–4.0 ppm associated with methoxy groups of FA (Fig 6A).

## Discussion

### Properties of two-domain laccase

The cloned bacterial laccase designated as SpSL contains two cupredoxine domains, characteristic of two-domain laccases [15, 45]. Two cupredoxin domains distinguishes two-domain laccases from three-domain ones, which contain three cupredoxin domains in the sequence [46]. SpSL contains the copper-binding motifs typical for laccases (histidines and one cysteine) [47]. The TAT signal peptide present in SpSL indicates that it is an extracellular enzyme. Secretion is characteristic of both two-domain and three-domain laccase [48, 49]. SpSL is a trimeric protein like other two-domain laccases reported previously [17, 48], although there is a report on the octameric enzyme [33]. The absorption maximum at 600 nm in the SpSL spectrum is due to the presence of the T1-copper center, while a shoulder at 340 nm is due to the presence of the T3-copper center in the enzyme. The high thermal stability of SpSL, its alkaline pH optimum and stability in the alkaline pH region (Fig 2) are typical for two-domain laccases and were reported for this type of laccase before [16, 17].

### Interaction of SpSL with low molecular weight substrates and humic acid in the presence of phenolic acids

Low molecular weight compounds can act as mediators of laccase expanding its oxidative potential [2]. Here, we tested the effect of phenolic acids on the ability of SpSL to oxidize natural low-molecular compounds and HA at an alkaline pH. 3.4-Dimethoxybenzoic alcohol and p-hydroxybenzoic acid were not oxidized by two-domain laccase alone. Humic acids are polymerized by two-domain laccase [33], which was also the result of the present study (Fig 3). Three-domain fungal laccases can depolymerize HA at acidic pH—both directly [4, 31, 50] and in the presence of mediators [51]. Therefore, it is unknown whether phenolic compounds will facilitate HA decomposition by two-domain laccase, or they will they will modify HA structure in the other way.

We have found that the non-phenolic compound 3.4 DMBA and phenolic compound *p*-HDB were not oxidized by SpSL in the presence of phenolic acids, while both phenolic acids were completely oxidized. Thus, the intermediate products of FA and CA oxidation by SpSL have low oxidation potential and cannot serve as mediators in the transformation of 3.4 DMBA and *p*-HDB by the enzyme.

During the interaction of SpSL with HA in the presence of phenolic acids, no formation of low molecular weight products was observed, that is, no depolymerization of the HA occurred (Fig 4A and 4C). Thus, ferulic and caffeic acids did not promote degradation of HA by SpSL at alkaline pH. Each of the phenolic acids was oxidized and polymerized by the enzyme (Fig 4B and 4D). This was not unexpected, since caffeic and ferulic acids are polymerized by fungal laccases [52, 53]. Ferulic acid forms dimers (DiFA), trimers, tetramers and higher polymers via 8-5-, 8-O-4-, 8-8-, 5-5-, and 4-O-5 linkages as a result of oxidative coupling [54, 55]. The 8-5-coupled FA occur in noncyclic, decarboxylated and benzofuran forms. The 8-8-coupled FA also occur in three forms including tetrahydrofuran form [53]. Similar types of linkages are described for CA oligomers [56]. The analysis of ^1^H-NMR spectra of poly-FA and of HA-FA (Fig 6A) and the comparison of the signals with those published for diferulates [57] allowed for the identification of protons belonging to FA dilactone, 8-8-diferulic acid and trans-lactone acid (compounds 1, 2 and 4; [57]) among the products of FA oxidation by SpSL. Formation of FA dilactones during laccase-catalyzed coupling of FA is known from the literature [58]. Besides dimers, higher molecular weight products were formed from each of the phenolic acids, which is confirmed by gel filtration data (formation of fractions with 10–12 kDa and >80 kDa) and by a broadband background under the sharp signals of individual compounds on the ^1^H-NMR spectra of poly-FA and poly-CA (Fig 6). The IR spectroscopy data suggest the formation of quinones as oxidation products of CA, as revealed by a very strong peak at 1630 cm^-1^ in the spectrum of poly-CA (Fig 5B). The peaks at 1260–1210 and 1080 cm^-1^ in IR spectra of polyphenolic acids may indicate the formation of ethers.

When SpSL was mixed with HA in the presence of CA or FA, oxidation of the HA and phenolic acids occurred. The acidification of the reaction mixtures to pH<2 and analysis of the precipitates by IR and ^1^H-spectroscopy showed a high contribution of poly-FA and poly-CA into HA structure (Figs 5 and 6). The aromaticity of HA increased. This can be interpreted by the co-precipitation of phenolic acid polymers with HA and/or by their co-polymerization with HA. While the former certainly occurred to some extent, the results of gel-filtration strongly suggest the formation of co-polymers between phenolic acids and the components of HA. Products of higher molecular weight were formed during the interaction of SpSL with HA in the presence of FA or CA than during the interaction of SpSL with HA or with each of the phenolic acids (Figs 3 and 4). A two-fold increase in peak average molecular weight of HA low molecular weight fraction (from 10 to 20 kDa) in the presence of phenolic acids and SpSL is most likely a result of cross-coupling between the components of HA low molecular weight fraction in the presence of phenolic acids. The participation of FA in cross-coupling reactions is well known from the literature, for example the formation of ferulic acid oligomers with coniferyl alcohol [59], the formation of diferulate bridges between carbohydrates and glycoproteins in cell walls of higher plants catalyzed by laccases and peroxidases [60]. To the best of our knowledge, participation of phenolic acids in cross-coupling reactions of HA components in the presence of two-domain laccase has not been documented so far.

## Conclusions

We have shown that ferulic and caffeic acids cannot act as mediators in the oxidation of low molecular weight compounds 3,4-dimethoxybenzoic alcohol and *p*-hydroxybenzoic acid by SpSL at an alkaline pH. Phenolic acids also did not act as mediators of HA decomposition by SPSL. Both phenolic acids were self-polymerized to form dimers and higher polymers. In addition, they formed cross-polymers with humic acid, resulting in a two-fold increase of HA low molecular weight fraction (from 10- to 20 kDa) and an increase in the amount of HA high molecular weight fraction. The interaction of phenolic acids with HA in the presence of SpSL resulted in an increase of aromaticity of HA, as revealed by IR- and ^1^H-NMR spectroscopy data. The results of the study extend our knowledge on the transformation of natural substrates by two-domain bacterial laccases and on the potential role of the enzyme in SOM formation at alkaline pH values.

## Supporting information

S1 Enzyme properties (XLSX)

S1 Gel-filtration (XLSX)

S1 Raw images (PDF)

S1 Spectra (XLSX)
