# Supplementary figures and images for: Transformation of low molecular compounds and soil humic acid by two domain laccase of Streptomyces puniceus in the presence of ferulic and caffeic acids
Source: PLoS One. 2020 Sep 18;15(9):e0239005. doi: 10.1371/journal.pone.0239005 (PMC7500650; doi:10.1371/journal.pone.0239005)

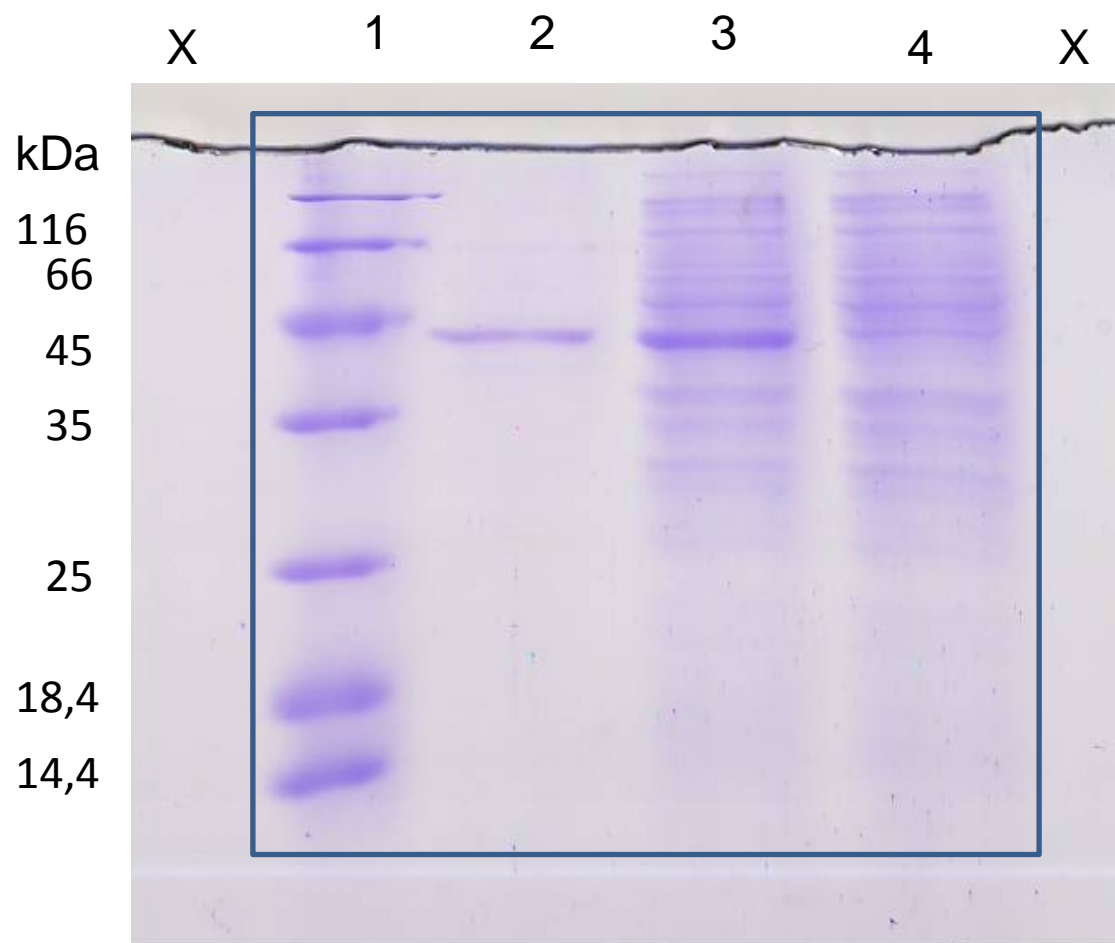

Supplement: S1 Raw images — (PDF) [file pone.0239005.s003.pdf]
